# Rapidly Progressive Merkel Cell Carcinoma with Atypical Metastatic Pattern—A Case Report and Brief Literature Review

**DOI:** 10.3390/diagnostics15232941

**Published:** 2025-11-21

**Authors:** Teodora Gabriela Alexescu, Denisa Lungu, Tania Popescu, Mirela Georgiana Perne, Cezara Andreea Gerdanovics, Olga Hilda Orășan, Angela Cozma, Ioana Raluca Dobrotă, Răzvan Dan Togănel, Mircea Vasile Milaciu, Lorena Ciumărnean, Nicoleta Valentina Leach, Călin Vasile Vlad, Vlad Alexandru Zolog, Cornel Dragoș Cheregi

**Affiliations:** 1 4th Medical Discipline, Department of Internal Medicine, “Iuliu Hațieganu” University of Medicine and Pharmacy, Republicii Street, No. 18, 400015 Cluj-Napoca, Romania; teodora.alexescu@umfcluj.ro (T.G.A.);; 2 Department of Neurology, County Emergency Hospital Cluj, Victor Babes Street, No. 43, 400012 Cluj-Napoca, Romania; 3 Department of Internal Medicine, Regional Institute of Gastroenetrology and Hepatology “Prof. Dr. Octavian Fodor”, Croitorilor Street, No. 19, 400394 Cluj-Napoca, Romania; 4 Department 2, Faculty of Nursing and Health Sciences, “Iuliu Hațieganu” University of Medicine and Pharmacy, Republicii Street, No. 18, 400015 Cluj-Napoca, Romania; 5 Faculty of Medicine, University of Medicine and Pharmacy Oradea, 1 Decembrie Plaza, No. 10, 410073 Oradea, Romania

**Keywords:** Merkel, cell, carcinoma, rare, metastatic, splenic, rapid, evolution, case report

## Abstract

**Background and Clinical Significance:** Merkel cell carcinoma (MCC) is a rare, aggressive neuroendocrine cutaneous malignancy with increasing incidence among elderly, immunocompromised patients or individuals exposed to ultraviolet radiation. **Case Presentation:** We present the case of an 84-year-old Caucasian male with no history of immunosuppression, who was admitted for asthenia, dysphagia, weight loss, and generalized weakness. Clinical and imaging investigations revealed a violaceous tumor on the right arm and disseminated metastases affecting the liver, spleen, bones and lymph nodes. A liver biopsy confirmed a small round blue cell neoplasm suggestive for MCC, although immunohistochemistry could not be performed due to the patient’s fulminant deterioration and death within 12 days of admission. **Conclusions:** This case is notable for its exceptionally rapid progression, particularly splenic involvement, and absence of known immunosuppressive factors. It highlights the existence of highly proliferative MCC subtypes with potential for bypassing classical metastatic pathways. Early clinical suspicion and prompt histological evaluation are essential for diagnosis, although the prognosis remains poor in advanced stages. Due to fulminant deterioration, immunohistochemistry could not be performed; therefore, the diagnosis is highly suggestive based on clinical, imaging, and morphological correlation.

## 1. Introduction

Merkel cell carcinoma (MCC) is a rare and highly aggressive neuroendocrine cutaneous malignancy, initially identified in 1972 by Cyril Toker under the name “trabecular carcinoma of the skin” [1]. The term “Merkel cell carcinoma” was adopted later because the tumor cells are histologically similar to Merkel cells—specialized cells located in the basal layer of the epidermis, particularly near hair follicles. Merkel cells function as mechanoreceptors for light and are linked to afferent sensory nerves. They exhibit neuroendocrine characteristics and express specific markers such as chromogranin A, synaptophysin and cytokeratin 20 (CK20—also known as keratin, type I cytoskeletal 20) [2,3]. These same markers are typically expressed by MCC cells as well. However, the origin of this cancer is controversial. Other studies have proposed that the tumor may originate from totipotent stem cells in the dermis or from precursor B cells [1,4].

The literature reports diverse causes and mechanisms underlying MCC development. Risk factors for Merkel cell carcinoma (MCC) include advanced age, fair skin, immunosuppression, significant sun exposure and the presence of other malignancies such as multiple myeloma or chronic lymphocytic leukemia (CLL) [4,5]. Most MCC cases are linked to the monoclonal integration of Merkel cell polyomavirus (MCPyV)—a non-enveloped, double-stranded DNA virus—into the host genome [5]. Although MCPyV is widespread, with antibodies found in approximately 80% of individuals over the age of 50, MCC is the only cancer known to be associated with it. In contrast, MCPyV-negative MCC cases are primarily driven by ultraviolet (UV)-induced DNA mutations [4].

On physical examination, MCC typically presents as a rapidly growing, firm, non-tender, violaceous nodule [6]. Clinically, Merkel cell carcinoma may present as a cutaneous or subcutaneous nodule, occasionally resembling a cyst. Lesions can appear in a range of colors, including red, pink, blue, violet or skin-toned. They are typically solitary and painless in the early stages but may ulcerate or develop surrounding satellite lesions. At the time of diagnosis, lesions generally measure less than 20 mm, though sizes can vary and most cases exhibit rapid tumor growth over the course of a few months [7]. The acronym AEIOU described by Heath et al. in 2008 has been used to summarize the most common clinical features: asymptomatic, expanding rapidly, immunosuppression, older than 50 and location on a UV-exposed site [6]. Histologically, MCC is categorized into three subtypes: trabecular, intermediate and small cell [8,9]. MCC lesions most commonly develop on sun-exposed areas, although approximately 19% occur on the buttocks or other regions with minimal sun exposure. The most frequent primary anatomical locations are the head and neck (29%), followed by the lower limbs (24%) and upper limbs (21%) [6,7].

MCC remains a highly aggressive neuroendocrine skin cancer, with a case-fatality rate exceeding that of melanoma. Over one-third of patients ultimately die of the disease. Notably, approximately one-third of cases already present with loco-regional metastases at the time of diagnosis, such as in-transit metastases—a tumor that is distinct from the primary lesion, located either along the lymphatic drainage pathway between the primary site and the regional lymph nodes, or beyond the primary site in a distal location—or with nodal involvement [10,11].

Since its initial description by Toker in 1972, the incidence of MCC has risen, a trend that has still continued into the 21st century. The rising proportion of individuals over 65 years of age, combined with persistently elevated levels of solar ultraviolet (UV) exposure worldwide, is anticipated to contribute to a continued increase in the incidence of skin cancers, including Merkel cell carcinoma [12,13,14].

In this context, we present the case of an elderly patient with metastatic Merkel cell carcinoma who experienced an unusually rapid clinical progression and had a fulminant evolution. Given the rarity and aggressive nature of MCC, this case highlights the aggressive course of MCC, the challenges of timely recognition and serves as a starting point for a broader discussion on similar presentations described in the literature.

## 2. Case Report

We report the case of an 84-year-old Caucasian male from a rural area, retired and with no history of smoking or alcohol use. He presented to the Internal Medicine IV Clinic at the CF Cluj-Napoca University Hospital with complaints of severe asthenia, dysphagia, loss of appetite, weight loss of 10 kg over the past month, intense back pain and generalized muscle weakness.

His medical history included significant cardiovascular comorbidities: newly diagnosed atrial fibrillation, chronic ischemic heart disease, NYHA class III heart failure, grade I arterial hypertension, and benign prostatic hyperplasia.

**Figure 1:**
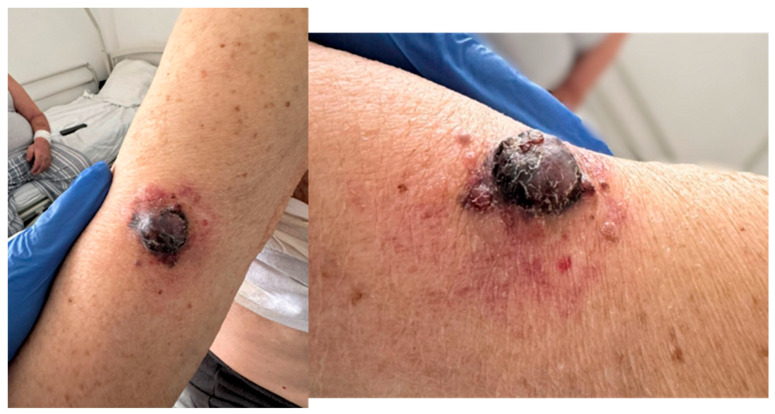
The patient had markedly enlarged right axillary lymph nodes and a red-violet, vegetative cutaneous lesion with satellite nodules and excoriation on the mid-anterior surface of the right arm.

On physical examination, the patient appeared pale and dehydrated, with hypomimic facies. Notably, he had markedly enlarged right axillary lymph nodes and a red-violet, vegetative cutaneous lesion (Figure 1) with satellite nodules and excoriation on the mid-anterior surface of the right arm. A contusion was also observed in the right pectoral region. The patient reported that the cutaneous lesion had been present for several months. The clinical context—lesion present for several months prior to evaluation, NYHA performance status ≥ 3 on admission and markedly elevated LDH—indicates a significant delay in seeking medical attention, which likely contributed to diagnosis at a preterminal stage and limited the feasibility of further diagnostic and therapeutic interventions.

Laboratory investigations revealed anemia, thrombocytopenia, leukocytosis with neutrophilia, elevated hepatic transaminases, hyperbilirubinemia, hypoalbuminemia, severely increased LDH and CRP, impaired renal function (eGFR 38.4 mL/min/1.73 m^2^), hypofibrinogenemia, and markedly elevated D-dimer levels. A summary of the test results is provided in Table 1.

Abdominal ultrasound revealed a globally enlarged liver with a multinodular, heterogeneous pattern and mild splenomegaly. Both kidneys were enlarged, with a polar cyst of 3.16 cm on the left side and multiple cysts on the right side, the largest measuring 4.3 cm. These findings raised suspicion of disseminated malignancy, and a liver biopsy was scheduled on day 4 of admission. The decision to perform a liver biopsy was made after thorough multidisciplinary evaluation and was considered clinically justified despite the patient’s systemic frailty. The decision to prioritize liver biopsy instead of cutaneous biopsy was based on the need for a rapid histopathological diagnosis of the visceral disease, given the patient’s rapidly deteriorating condition and multiple hepatic nodules on imaging. Liver biopsy could be performed immediately under ultrasound guidance at bedside, while cutaneous biopsy required dermatologic consultation and was not feasible in an emergency context. Laboratory testing before the procedure demonstrated normal coagulation parameters, with an INR of 1.27 and fibrinogen level of 142.2 mg/dL, values acceptable for minimally invasive liver biopsy according to standard procedural safety thresholds (INR < 1.5). Platelet count was 108 × 10^9^/L, which, although slightly decreased, remained within the range considered safe for percutaneous biopsy under ultrasound guidance. According to hepatology and interventional radiology standards (platelets ≥ 50 × 10^9^/L and INR ≤ 1.5), these values are considered acceptable for ultrasound-guided liver biopsy [15]. Therefore, the decision to proceed with the biopsy was appropriate and in accordance with international hepatology and oncology practice standards.

Due to progressive dysphagia for solids, an upper GI endoscopy was also performed on the fourth day of admission, showing Los Angeles grade B esophagitis, erosive pangastritis and a subcardial polyp.

**Figure 2:**
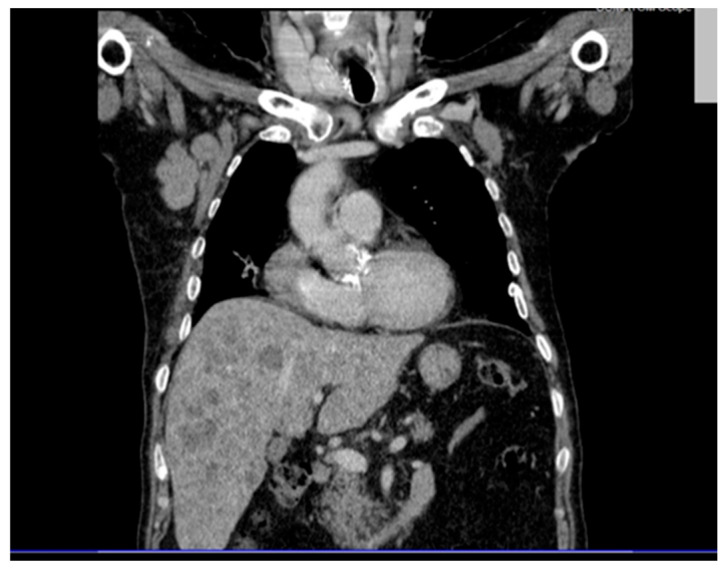
Contrast-enhanced thoracic CT scan showing pleural thickening up to 9 mm in the posterior segment of the right lower lobe and along the right oblique fissure, as well as a linear atelectasis in the middle lobe. Enlarged right axillary lymph nodes are also noted, measuring up to 32 × 41 mm.

To further investigate, a contrast-enhanced and non-contrast CT scan of the thorax, abdomen, and pelvis was carried out, with the following pathological findings:

Thoracic CT (contrast and non-contrast) (Figure 2) revealed pleural thickening up to 9 mm posterior to the right lower lobe and along the right oblique fissure extending towards the left upper lobe, along with a band of atelectasis in the middle lobe. Significantly enlarged right axillary lymph nodes were observed, measuring up to 32 × 41 mm.

**Figure 3:**
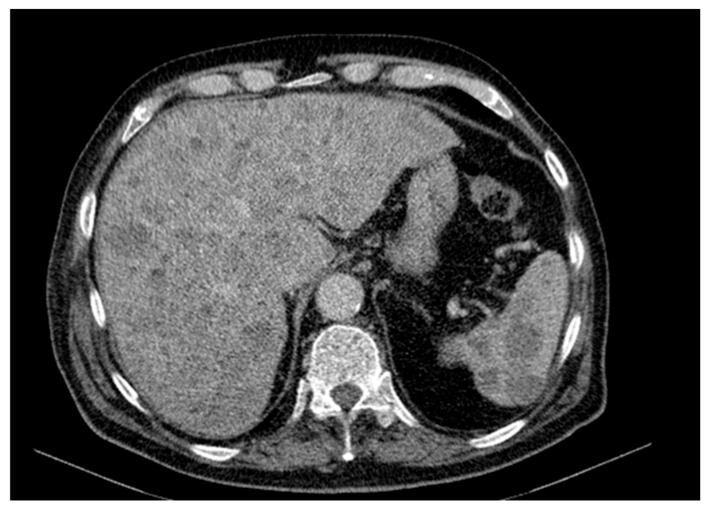
Contrast-enhanced abdominal and pelvic CT scan revealing hepatosplenomegaly with multiple hypodense, non-enhancing nodular lesions suggestive of secondary involvement. Bilateral renal cysts, more numerous and larger on the right (up to 50 mm), a 9 mm right renal pelvis stone, and the presence of a right ureteral stent are noted. Diffuse bone structure changes with osteoporotic background and poorly defined hyperdensities are seen, along with L1 vertebral body collapse.

Abdominal and pelvic CT revealed hepatosplenomegaly with multiple hypodense, non-enhancing nodular lesions, precaval and hilar lymphadenopathy, bilateral renal cysts (up to 50 mm on the right), a right renal pelvis stone, a right ureteral stent and diffuse osteoporotic changes with vertebral L1 collapse and hyperdense nodular lesions suggestive of possible bone metastases (Figure 3).

Clinical deterioration occurred approximately two hours after the liver biopsy, with hypotension (BP = 65/40 mmHg), tachycardia (HR = 125 bpm), SpO_2_ = 92%, profuse sweating, nausea and marked pallor. A hemoperitoneum was diagnosed on emergent abdominal ultrasound and peritoneal drainage was performed. The patient was transferred to the ICU, where he required vasopressor support (Noradrenaline) and oxygen therapy on nasal cannula. The post-biopsy hemoperitoneum further destabilized the patient, aggravating an already fragile and preterminal clinical condition and eliminating the possibility of additional diagnostic or therapeutic measures.

Additional laboratory investigations such as HIV/HBV/HCV serology, immunoglobulin levels, lymphocyte subpopulations, protein electrophoresis and tumor markers (chromogranin A, NSE) were planned, but could not be performed because of the patient’s fulminant deterioration and transfer to the ICU shortly after liver biopsy.

Upon admission to the ICU, a new abdominal ultrasound revealed blood-tinged fluid in the abdominal cavity, suggesting a hemoperitoneum secondary to the liver biopsy. Ultrasound-guided peritoneal drainage was performed. Despite intensive supportive care, his clinical condition further worsened, with jaundice, bilateral pulmonary rales, abdominal tenderness in the right lower abdomen and hypogastrium, rising inflammatory markers, persistent anemia and coagulopathy. Laboratory results showed persistent inflammatory syndrome (CRP = 65 mg/L), azotemia and markedly elevated D-dimer levels. Repeated transfusions achieved a Hb level of 8.8 g/dL. The patient developed atrial fibrillation with a rapid ventricular rate, refractory to conversion therapy and continuous antiarrhythmic infusion, followed by progressive neurological decline to a Glasgow Coma Scale score of 7–8. On day 12 of hospitalization, the patient suffered a cardiorespiratory arrest and resuscitation efforts were unsuccessful.

**Figure 4:**
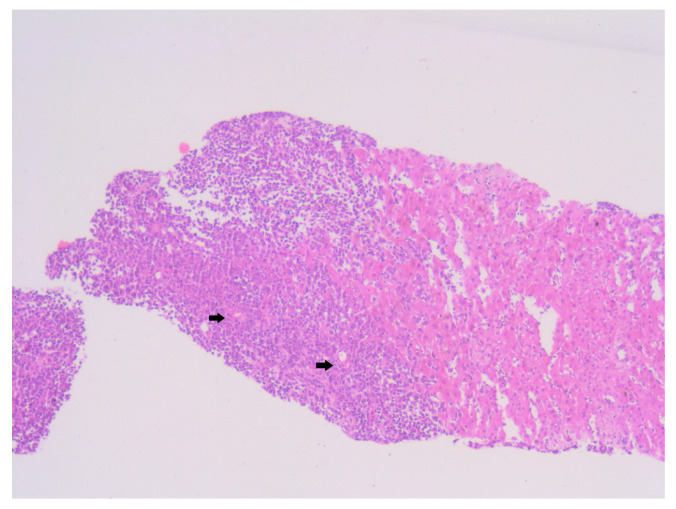
Hematoxylin–Eosin staining, 4× magnification (HE 4×): Biopsied fragment of hepatic parenchyma showing a tumoral proliferation located at one end, occupying approximately three high-power fields (HPFs). Arrows indicate the area of tumoral infiltration at the edge of the hepatic parenchyma.

**Figure 5:**
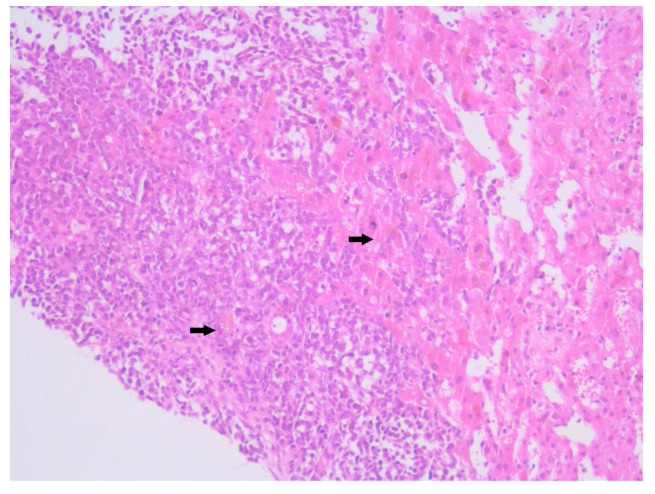
Hematoxylin–Eosin staining, 10× magnification (HE 10×): Tumoral proliferation composed of diffusely arranged cells forming sheets of variable sizes, separated by minimal stroma. Arrows highlight clusters of small round blue cells arranged in diffuse sheets.

**Figure 6:**
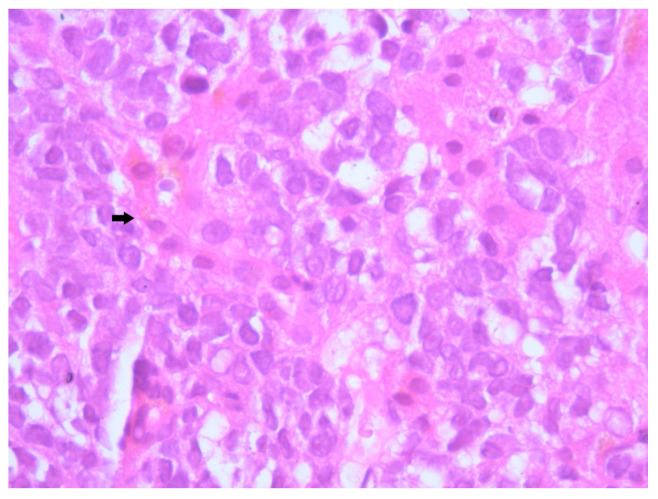
Hematoxylin–Eosin staining, 40× magnification (HE 40×): The neoplastic cells are small, round, with indistinct cellular borders, minimal eosinophilic cytoplasm, and round to oval nuclei that are either hyperchromatic or euchromatic, with homogeneous chromatin, inconspicuous nucleoli, and rare mitotic figures. Arrows indicate representative neoplastic cells with scant cytoplasm and finely granular chromatin.

Histopathological examination of the liver biopsy revealed fragments of hepatic parenchyma measuring 10 × 1 mm, with tumoral infiltration by small, round blue cells exhibiting indistinct cytoplasmic borders, scant eosinophilic cytoplasm and round to oval nuclei that were either hyperchromatic or euchromatic, with finely granular chromatin and absent nucleoli. Occasional mitotic figures (4 per 3 HPFs) were identified. The morphology was consistent with a small round blue cell tumor, raising differential diagnostic considerations (Figure 4, Figure 5 and Figure 6). Small round blue cell tumors involving the liver include lymphoma, plasmacytoma, metastatic small-cell lung carcinoma, high-grade neuroendocrine carcinoma, metastatic melanoma, metastatic neuroblastoma and Ewing sarcoma. In contrast to these entities, MCC usually presents with a rapidly growing cutaneous primary tumor, satellite or in-transit metastases and early involvement of regional lymph nodes, as seen in our patient. The combination of morphology (small round blue cells with scant cytoplasm and neuroendocrine features), the presence of a violaceous primary lesion on a sun-exposed site, satellite nodules, marked axillary lymphadenopathy and rapid multiorgan dissemination made MCC the most consistent diagnosis, despite the absence of immunohistochemistry. To clarify the cellular origin and reach a definitive diagnosis, correlation with clinical and paraclinical data, along with immunohistochemical testing (e.g., CK AE1/AE3, CK20, CD56, Chromogranin A, SOX10, CD20, CD3, CD138), would have been essential. The liver biopsy was obtained while the patient was hemodynamically unstable, and within hours he developed biopsy-related hemoperitoneum, shock and multiorgan failure, requiring transfer to the ICU. Due to the rapid clinical deterioration and death shortly afterward, the sample could not be processed for IHC in time, and further invasive tissue sampling was no longer ethically indicated. Nevertheless, the combination of clinical presentation (violaceous cutaneous tumor with satellite nodules), imaging (multiorgan metastases) and morphology of the liver sample (small round blue cells with neuroendocrine features) supported the presumptive diagnosis of metastatic MCC. According to the 8th edition of the American Joint Committee on Cancer (AJCC) staging system for MCC, this case corresponds to Stage IV—cT2N3M1c disease. The T2 classification applies because the primary cutaneous tumor was >2 cm. N3 reflects the presence of clinically evident, macroscopic lymph node involvement together with in-transit metastases (satellite cutaneous nodules). The M1c designation is supported by multiple visceral metastases involving the liver, spleen and suspected bone lesions on CT imaging.

## 3. Discussion

Merkel cell carcinoma (MCC) poses significant diagnostic and therapeutic challenges due to its rarity, aggressive course and potential for early metastasis. While the clinical and pathological characteristics of MCC have been increasingly documented in recent years, cases with fulminant evolution and atypical metastatic patterns remain uncommon and poorly understood. In this section, we compare the clinical, pathological and epidemiological features of our case with the existing literature, highlighting both shared and distinct elements. Particular attention is given to the rapidity of disease progression, the unusual distribution of metastases and potential contributing factors such as age, comorbidities, immune status and environmental factors.

Ultraviolet (UV) radiation is recognized as the primary risk factor for skin cancers, particularly in individuals with fair skin. It plays a major role in the development of both keratinocyte carcinomas and melanoma, and growing evidence suggests its involvement in Merkel cell carcinoma (MCC) as well. This is supported by the higher incidence of MCC in regions with elevated UV radiation levels and its frequent localization on sun-exposed areas such as the head and neck [7,14].

In addition to UV exposure, environmental heat may also contribute to skin carcinogenesis. Experimental studies on cell cultures and animal models have shown that repeated exposure to UVB radiation combined with heat (around 39 °C) impairs cellular apoptosis by suppressing the p53-mediated stress response and increasing the expression of sirtuin 1 (SIRT1). These environmental factors may therefore act synergistically to promote the survival of cells with DNA damage [14]. In this context, the patient in our case, being an agricultural worker for a long time, was chronically exposed to sunlight and high temperatures. This occupational exposure could contribute to his elevated risk of developing MCC. In rural areas in Romania, the use of sunscreen is uncommon among elderly individuals, further amplifying the risk in populations with prolonged outdoor activity and limited photoprotection.

In addition to the well-established risk factors for MCC—advanced age, UV radiation exposure, immunosuppression and MCPyV infection—environmental factors have been suggested as possible contributors. Some studies have explored whether chronic exposure to agricultural chemicals, including pesticides, might play an indirect role in skin carcinogenesis. These associations have been documented more consistently for melanoma and non-melanoma skin cancers, while evidence for MCC remains anecdotal and limited to isolated case reports. Therefore, the relationship should be regarded as a hypothesis rather than a confirmed risk factor, particularly in populations with prolonged outdoor occupational exposure [16,17,18]. While only a limited number of Merkel cell carcinoma (MCC) cases (14 in total) have been directly linked to arsenic exposure [19,20], these findings raise the broader possibility that MCC, like melanoma, may also be influenced by chronic contact with agricultural chemicals.

To contextualize our case, we performed a focused literature search to identify previously published rapidly progressive or fulminant Merkel cell carcinoma (MCC) cases. A narrative search was conducted in PubMed, Scopus and Google Scholar using the following keywords: “Merkel cell carcinoma”, “fulminant”, “rapid progression”, “aggressive”, “metastatic pattern”, and “case report”. The search timeframe included articles published from January 2000 to January 2025. Only articles available in English and describing MCC cases with rapid clinical deterioration, multiorgan dissemination, or death within a short interval were included. Due to the rarity of such cases, the goal was not to perform a systematic review, but to summarize representative reports relevant to the clinical scenario of our patient (Table 2).

Although no direct epidemiological studies have confirmed a clear connection between MCC and general pesticide exposure, it is necessary to consider such a link, especially in occupational settings. In our case, the patient worked as a farmer for decades, likely encountering both arsenic residues and other pesticide compounds. This environmental exposure may represent an additional risk factor contributing to the development of MCC in this patient. While current evidence primarily supports ultraviolet radiation as the main environmental risk factor, isolated reports—such as the present case—highlight the need to explore additional environmental contributors. Prospective studies focusing on agricultural populations may offer valuable insights into how chronic exposure to pesticides and other farming-related factors might influence MCC risk.

MCC classically arises on sun-exposed regions—most commonly the head and neck (29%), followed by the lower limbs (24%) and upper limbs (21%) [6,7]. Our patient presented with a violaceous, vegetative lesion on the anterior surface of the right arm, a location consistent with the typical photo-exposed distribution. According to one study, upper limbs (24%) are the second most frequent site for MCC, after the head and neck (43%) [4]. Despite this, many lesions may be initially misinterpreted as benign vascular or inflammatory conditions, which may delay diagnosis, particularly in elderly patients with other comorbidities. Interestingly, when examining the fulminant cases, four of six also originated from limbs or extremities, a distribution consistent with the typical sun-exposed pattern but possibly reflecting a subset of MCCs with higher metastatic behavior. These cases suggest that tumors arising in peripheral regions may follow more unpredictable or systemic metastatic routes, sometimes bypassing the expected nodal pathway and leading to rapid multiorgan dissemination [20,21,23].

Recent dermoscopic studies have emphasized that early clinical suspicion of MCC may be improved through recognition of characteristic—although nonspecific—dermoscopic patterns. A recent retrospective analysis of histologically confirmed MCCs described four representative dermoscopic subtypes: the pinkish plaque type, cherry-red nodular type, ulcerated erythematous nodular type, and hyperkeratotic nodular type. These subtypes typically display structureless areas on a pinkish-white background (reported in 75% and 62.5% of cases, respectively), together with polymorphous vascular patterns including irregular linear and arborizing vessels. Although these features are not specific, their recognition may facilitate earlier identification of suspicious lesions and prompt histopathological confirmation. In our case, the violaceous, vegetative lesion on the sun-exposed surface of the right arm corresponds most closely to the “ulcerated erythematous nodular” dermoscopic subtype. The opalescent appearance and rich vascularization described in the literature were also clinically apparent, aligning with the hypothesis that MCC may initially mimic benign vascular or inflammatory conditions. Integrating dermoscopic assessment into initial evaluation protocols may therefore contribute to reducing diagnostic delay, particularly in elderly patients with atypical nodular or plaque-like presentations [25].

The demographic characteristics of our patient are consistent with epidemiological patterns described in large MCC cohorts. According to a study, the majority of MCC patients are non-Hispanic White (96.4%), male (62.6%) and over the age of 65 (82.1%), with a mean age at diagnosis of 74.5 years [11]. Another demographic study in the US by Paulson et al. revealed that approximately two-thirds of Merkel cell carcinoma cases have been diagnosed in men, a proportion that remained consistent between 2000 and 2013 [12]. Other study confirms that the highest incidence of patients (98%) affected by MCC are Caucasians [6]. Our patient, an 84-year-old Caucasian male, fits squarely within this demographic profile.

Also, when comparing the present case with the previously reported rapidly progressive MCCs, several converging trends emerge. The mean age among these fulminant cases was 63 years (range 38–84), slightly lower than the overall epidemiologic mean of approximately 74.5 years reported in large cohorts [11]. This suggests that younger patients are not necessarily protected against highly aggressive courses and that tumor biology, rather than age alone, may drive rapid dissemination.

MCC classically exhibits morphological features including small round cells with scant cytoplasm, nuclear molding, and hyperchromatic nuclei with finely granular “salt and pepper” chromatin—features reflective of its neuroendocrine differentiation [13]. In our patient, microscopic examination revealed a proliferation of small, undifferentiated cells with high nuclear-to-cytoplasmic ratio, frequent mitotic figures and dense nuclear chromatin, consistent with these hallmarks. Unfortunately, due to the fulminant clinical deterioration and subsequent death of the patient, immunohistochemical (IHC) profiling could not be completed. Nevertheless, the suggestive histological pattern in combination with the rapid evolution and metastatic behavior strongly supported the diagnosis of MCC. While IHC remains critical for definitive diagnosis, particularly to differentiate MCC from other small round blue cell neoplasms, the clinicopathological context provided sufficient grounds for a diagnosis.

Merkel cell carcinoma (MCC) is recognized for its aggressive behavior and high metastatic potential [11,12], yet the fulminant evolution observed in our patient remains exceptional in both rapidity and extent. In large cohorts, approximately 65–70% of patients present with localized disease at diagnosis (American Joint Committee on Cancer AJCC stage I–II), 25% with regional nodal involvement (AJCC stage III) and only 5–8% with distant metastases (AJCC stage IV) [26]. In contrast, our patient presented directly with widespread systemic involvement, including beyond lymph nodes, the liver, bones, and spleen at the time of diagnosis.

**Figure 7:**
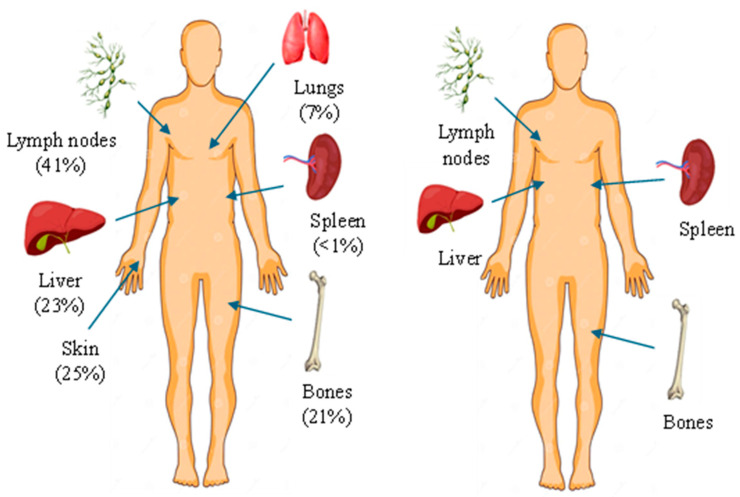
Anatomical distribution of metastases based on Lewis et al. [27] (*n* = 215 patients, Lewis et al. [27] vs. metastatic sites identified in the present case: liver, spleen, bone, lymph nodes), illustrating a pattern with splenic involvement and rapid multiorgan dissemination.

Consistent with a recent study, distant metastases in MCC most commonly affect lymph nodes (41%), skin/body wall (25%), liver (23%), bone (21%) and lungs (7%) [27]. Gastrointestinal tract involvement is rare, and splenic metastases are particularly uncommon, being reported in only ~1% of patients at initial distant progression (Figure 7) [27]. Our case therefore aligns only partially with established metastatic trends and highlights the spleen as an unusual—but documented—site of involvement.

When comparing the distribution of metastases in our patient with those reported in the fulminant cases, a striking overlap emerges. Among these previously described cases, visceral dissemination was almost universal, with the liver being the most frequently affected organ, followed by bone, brain, pleura and distant lymph nodes. Several reports even noted multifocal metastases at initial diagnosis, underscoring the capacity of this tumor subtype to disseminate rapidly and extensively. Despite anatomical heterogeneity of the primary lesion, these cases share a common pattern of early visceral spread, often in the absence of significant nodal involvement, suggesting a preferential hematogenous metastatic route rather than the classical stepwise lymphatic progression. This observation is further supported by the short survival times (ranging from a few days to a few months) described across the reviewed cases, indicating that the presence of multiple synchronous visceral metastases portends a near-uniformly fatal outcome.

Other studies also suggest that the site of the primary tumor may influence metastatic spread patterns. Lewis et al. reported that patients with MCC on the upper limbs were significantly more likely to develop distant skin or body wall metastases (43.5% vs. 20.1%, *p* = 0.002) compared to other primary sites and less frequently developed liver metastases [27]. In contrast, liver metastases were strongly associated with head and neck localisation (43.1% vs. 15.3%, *p* < 0.001), while lower limb primaries were associated with distant nodal involvement and lower liver dissemination rates [28].

The primary lesion was located on the anterior surface of the right arm, which would suggest a higher risk for cutaneous or superficial spread. However, this pattern was not observed. Instead, the patient presented with extensive visceral involvement, including the liver and spleen, bypassing the expected pathway of skin or nodal progression (Figure 4). This deviation may indicate either an aggressive tumor phenotype or possible hematogenous dissemination that override classical lymphatic pathways. The presence of both hepatic and splenic metastases, despite an upper limb origin, underlines an atypical evolution that contrasts with established metastatic models.

Because the spleen lacks afferent lymphatic vessels, splenic metastases occur exclusively via hematogenous dissemination. A recent review demonstrated that splenic involvement is observed almost exclusively in the setting of multiple synchronous visceral metastases, whereas isolated splenic metastasis is exceedingly rare. From an immunological perspective, the spleen contains a high density of immunocompetent and cytotoxic cells capable of eliminating circulating malignant cells. Experimental studies indicate that the splenic microenvironment favors pro-apoptotic signaling, actively promoting the destruction of tumor cells before they can develop into clinically detectable metastases. Therefore, even though circulating tumor cells may reach the spleen hematogenously, metastatic colonization rarely occurs because the splenic microenvironment is highly immunoreactive and inhospitable to tumor cell survival and proliferation. In our patient, the presence of splenic metastasis—despite an immunocompetent background—suggests an unusually aggressive hematogenous dissemination pattern capable of breaching the splenic immunologic barrier [29].

When compared with the fulminant MCC cases summarized in Table 2, similar metastatic tendencies can be observed. Moreover, several of the reviewed patients developed multiorgan metastases within weeks of diagnosis, and all experienced a fatal outcome within a short time frame, ranging from days to a few months, even when systemic therapy or radiotherapy was attempted.

Importantly, our patient had multiple metastatic sites at first presentation, a condition associated with significantly poorer outcomes. Also in the analysis by Lewis et al. [27], patients with multiple metastatic sites had a hazard ratio (HR) of 2.73 for MCC-specific mortality compared to those with a single site. Furthermore, liver metastases (HR = 2.13) and nodal metastases (HR = 1.97) were independently associated with worse prognosis [27]. Even in those cases, however, the median survival following diagnosis of distant metastasis was approximately 359 days, with 1-year survival of 45% [28]. While the literature acknowledges MCC’s rapid course, the fulminant evolution observed in our patient, culminating in death within 12 days of hospital admission, represents a highly atypical evolution. In our case, survival was very limited, emphasizing the fulminant behavior of this tumor rarely described in literature.

Bone metastases in Merkel cell carcinoma (MCC) are increasingly recognized as part of the metastatic spectrum and may take osteolytic, osteoblastic, or mixed forms. A recent multicenter radiologic analysis by Guadagnolo et al. [30] reported that bone involvement occurs in approximately 21% of patients with distant metastatic MCC. The study highlights that MCC osseous metastases often demonstrate a permeative or infiltrative growth pattern, sometimes associated with cortical destruction or vertebral collapse—findings consistent with the imaging appearance in our patient (L1 vertebral body collapse and hyperdense nodular lesions on CT) [30]. Although osteolytic lesions are more frequently described, mixed or sclerotic patterns may also occur, complicating differentiation from metastatic prostate or breast cancer. Importantly, patients with bone metastases have significantly poorer survival compared to those with soft-tissue or nodal-only progression, suggesting that osseous dissemination reflects a biologically aggressive subtype of MCC. In our case, the suspected vertebral metastasis aligned with the fulminant clinical deterioration and widespread visceral involvement, supporting the hypothesis that rapid hematogenous spread drives the aggressive trajectory observed.

In general, Merkel cell carcinoma exhibits rapid growth, with most tumors doubling in size within a matter of weeks to a few months [6]. Notably, tumor doubling times have been reported to range between 5 and 12 days and in the aggressive subtypes, 1 to 5 days [28]. This aggressive tumor pattern may explain the rapid clinical decline in our patient who developed widespread metastases and died in less than two weeks after hospital admission. Such a fulminant course is suggestive of a highly proliferative subtype of MCC, despite the absence of confirmed immunosuppression or hematologic malignancy. This case supports the concept that a subset of MCC tumors possess intrinsic hyperproliferative and dissemination potential, capable of bypassing expected metastatic stages and causing fatal systemic involvement within a matter of days.

Most patients undergo multimodal therapy including surgery, radiotherapy and immunotherapy. Despite surgical or immunotherapeutic interventions in some reports summarized in Table 2, all cases culminated in rapid deterioration and death within days to months, confirming that this subset of MCC demonstrates resistance to conventional multimodal therapy and extremely short survival times. In contrast with the median survival of approximately 12 months typically reported for stage IV disease [27,28], the mean survival across the fulminant cases presented in Table 2 was only around 2–3 months, underscoring the aggressiveness of this variant. Notably, our patient exhibited the most severe course, with death after only 12 days of hospitalization, representing one of the shortest survivals documented in the literature. Although coagulation parameters met guideline safety thresholds, the patient developed a post-biopsy hemoperitoneum, illustrating that clinically acceptable laboratory values do not entirely eliminate bleeding risk in fragile, systemically unstable patients. Also, the patient’s condition deteriorated so quickly that oncologic treatment could not be started, showing how severe and unusually fast the disease progression was.

## 4. Limitations

A significant limitation of this case is the absence of immunohistochemical (IHC) confirmation of the suspected diagnosis. Although the histopathological evaluation of the liver lesion did not include IHC staining, the morphological and clinical context provided strong diagnostic evidence of Merkel cell carcinoma. The patient was hospitalized in an internal medicine ward, where urgent diagnostic and supportive care were prioritized due to rapid systemic deterioration. At the time of biopsy, referral to oncology for IHC confirmation was already planned. According to the institutional workflow in our hospital, immunohistochemistry (IHC) requests can only be initiated by an oncology specialist, which requires the patient to first be discharged from our internal medicine service and formally admitted to the oncology department for registration and requisition of the IHC tests. Given the patient’s critical and rapidly deteriorating condition, discharge and transfer were clinically unsafe and not feasible. Consequently, IHC processing could not be initiated in time. 

A biopsy of the primary cutaneous lesion was not feasible post-mortem, as the diagnosis of MCC had already been established morphologically on liver tissue and confirmed clinically by the characteristic cutaneous presentation (violaceous nodular lesion with satellite nodules and rapid growth). Given the patient’s terminal status and the absence of therapeutic impact, further invasive procedures were not ethically justified.

Nonetheless, the combination of morphological features (small round blue cells with neuroendocrine morphology), the clinical appearance of the cutaneous lesion and the fulminant systemic progression strongly supported the presumptive diagnosis of metastatic Merkel cell carcinoma, consistent with the criteria described in the literature for rapidly progressive MCC in elderly immunocompetent patients.

## 5. Ethical Approval and Informed Consent

The patient described in this report was deceased at the time of manuscript preparation. Therefore, written informed consent for publication could not be obtained directly. However, the case was reviewed and approved for publication by the institutional ethics committee of the Cluj-Napoca CF Hospital, in accordance with the ethical standards of the Declaration of Helsinki and local regulations governing retrospective case reports. All identifying information has been removed to ensure full patient anonymity.

## 6. Conclusions

Although advanced-stage MCC is not uncommon at diagnosis, especially in elderly patients with comorbidities, this case is remarkable due to its fulminant evolution, with multiorgan dissemination and death within only 12 days of hospital admission. The delayed medical consultation (lesion present for several months, NYHA PS ≥ 3 and markedly elevated LDH) likely contributed to diagnosis in a preterminal setting, a scenario frequently encountered in MCC.

Unlike the expected progression of MCC, which often begins with localized skin involvement and sequential nodal and cutaneous dissemination, our patient presented directly with advanced multiorgan metastases, including the liver, bone and notably the spleen—an exceedingly rare site of involvement. The absence of an immunocompromised background, along with the extremely short tumor doubling time inferred from the clinical evolution, suggests an aggressive tumor biology.

This case emphasizes the importance of early recognition of MCC, particularly in elderly patients with cutaneous nodular lesions, but also illustrates that once regional spread occurs (satellite lesions and axillary lymph-node involvement), systemic dissemination may progress extremely rapidly, resulting in fatal outcomes within days. Increasing awareness of MCC among general practitioners and geriatric physicians is essential, as early biopsy and referral may allow curative treatment in localized disease. Since MCC may initially mimic benign vascular or inflammatory lesions, prompt recognition and timely dermatologic or oncologic evaluation can significantly improve survival outcomes. Also, while UV radiation remains the most clearly established environmental driver of MCC, the possible role of chronic pesticide exposure, particularly in agricultural workers, warrants further investigation. This case raises important questions and may serve as a starting point for future research exploring environmental and occupational risk factors for MCC in rural and farming populations.

## Figures and Tables

**Table 1:** Relevant Laboratory Parameters.

Parameter	Patient Value	Reference Range
Hemoglobin (Hb)	10.4 g/dL	13.5–17.5 g/dL (male)
White Blood Cells (WBC)	12.10 × 10^9^/L	4.0–10.0 × 10^9^/L
Neutrophils	9.25 × 10^9^/L	1.5–8 × 10^9^/L
Lymphocytes	2.15 × 10^9^/L	1.5–4.0 × 10^9^/L
Platelets (PLT)	108 × 10^9^/L	150–400 × 10^9^/L
AST (SGOT)	195 U/L	0–50 U/L
ALT (SGPT)	235 U/L	0–50 U/L
Total Bilirubin	2.55 mg/dL	0.1–1.2 mg/dL
Serum Albumin	3.30 g/dL	3.5–5.2 g/dL
LDH	1613 U/L	135–225 U/L
CRP	37.67 mg/L	<5 mg/L
Urea	88.5 mg/dL	16.6–48.5 mg/dL
Serum Creatinine	1.63 mg/dL	0.7–1.2 mg/dL
eGFR (CKD-EPI)	38.4 mL/min/1.73 m^2^	>90 mL/min/1.73 m^2^
INR	1.27	0.8–1.5
Prothrombin Time (PT)	21.1 s	14.3–18.3 s
Fibrinogen	142.2 mg/dL	180–450 mg/dL
D-dimers	23,734 ng/mL FEU	<500 ng/mL FEU

**Table 2:** Summary of reported cases of Merkel cell carcinoma with fulminant progression.

Author (Year)	Age/Sex	Primary Site	Imaging	IHC Confirmation	Immunological Status	Metastatic Sites	Clinical Course	Outcome
Present case	84/M	Mid-anterior surface of the right arm	CT	No	Immunocompetent	Liver, spleen, bone	Fulminant progression despite hospitalization and supportive care	Died after 12 days of hospitalization
Yaghmour et al., 2024 [21]	55/F	Right upper thigh	CT/MRI	Yes (CK20+, Synaptophysin+, Ki67 ~70%)	Immunocompetent	Brain	Rapid deterioration—palliative treatment	-
Caldarelli et al., 2019 [19]	82/F	Forehead	AngioCT	Yes (CK20+, Chromogranin+, neuroendocrine pattern)	Immunocompetent	Parotid	Stable after surgery but refused RT Rapid deterioration	Died from the illness
Liu et al., 2023 [20]	38/M	Left index finger	-	Yes (CK20+, neuroendocrine morphology)	Immunocompetent	Liver	Rapid deterioration	Died one year after initial diagnosis
Jain et al., 2025 [22]	66/M	Right thigh	CT	Not reported	Immunocompetent	Mass encased the aortic arch, left common carotid artery and left subclavian artery	Palliative care—rapid deterioration	Died after one month
Oliveira et al., 2025 [23]	77/F	Left lateral cervical region	CT	Yes (CK20+, CK7−, neuroendocrine markers+)	Immunocompetent	Solid mass with no distinct cleavage plane separating it from the anterior surface of the sternocleidomastoid muscle or the ascending ramus of the left mandible	Immunotherapy with Avelumab—the mass exhibited progressive growth, culminating in skin fistulization and associated hemorrhage and rapid deterioration with a severe acute respiratory syndrome	Died at 6 months
McNulty et al., 2024 [24]	59/F	Breast	PET-CT	Yes (CK20+, CAM5.2+, Synaptophysin+, Chromogranin+, Ki67 > 90%)	Immunocompromised (autoimmune disease on immunomodulatory therapy)	Lymph nodes, pleura, liver, bones, pancreas and left lateral thigh soft tissue	Rapid decline requiring intubation and pressors	Died less than 3 weeks after the PET-CT

## Data Availability

The original contributions presented in this study are included in the article. Further inquiries can be directed to the corresponding authors.

## Abbreviations

The following abbreviations are used in this manuscript:

AJCC	American Joint Committee on Cancer
ALT	Alanine Aminotransferase
AST	Aspartate Aminotransferase
CK20	Cytokeratin 20
CRP	C-reactive Protein
CT	Computed Tomography
eGFR	Estimated Glomerular Filtration Rate
GI	Gastrointestinal
Hb	Hemoglobin
HBV	Hepatitis B Virus
HCV	Hepatitis C Virus
HIV	Human Immunodeficiency Virus
HPF	High Power Field (microscopy)
IHC	Immunohistochemistry
ICU	Intensive Care Unit
INR	International Normalized Ratio
LDH	Lactate Dehydrogenase
MCC	Merkel Cell Carcinoma
MCPyV	Merkel Cell Polyomavirus
NYHA	New York Heart Association (classification of heart failure)
PLT	Platelets
PT	Prothrombin Time
TNF-α	Tumor Necrosis Factor Alpha
WBC	White Blood Cells
